# The latest shallow-sea isocrinids from the Miocene of Paratethys and implications to the Mesozoic marine revolution

**DOI:** 10.1038/s41598-024-67687-2

**Published:** 2024-08-02

**Authors:** Mariusz A. Salamon, Urszula Radwańska, Karolina Paszcza, Marcin Krajewski, Tomasz Brachaniec, Robert Niedźwiedzki, Przemysław Gorzelak

**Affiliations:** 1 Faculty of Earth Sciences, Laboratory of Palaeontology and Stratigraphy, University of Silesia in Katowice, Będzińska 60, 41-200 Sosnowiec, Poland; 2 Faculty of Geology, Department of Historical, Regional Geology and Paleontology, University of Warsaw, Żwirki i Wigury93, 02-089 Warszawa, Poland; 3 Doctoral School at the University of Silesia in Katowice, Bankowa 14, 40-007 Katowice, Poland; 4 Faculty of Geology, Geophysics and Environmental Protection, AGH University of Science and Technology, al. A. Mickiewicza 30, 30-059 Kraków, Poland; 5 Faculty of Earth Sciences and Environmental Management, Institute of Geological Sciences, Wrocław University, Cybulskiego 30, 50-205 Wrocław, Poland; 6 Institute of Paleobiology, Polish Academy of Sciences, Twarda 51/55, 00-818 Warsaw, Poland

**Keywords:** Echinoderms, Sea lilies, Predation, Neogene, Cenozoic, Palaeontology, Evolutionary ecology

## Abstract

The predation-driven Mesozoic marine revolution (MMR) is believed to have induced a dramatic change in the bathymetric distribution of many shallow marine invertebrates since the late Mesozoic. For instance, stalked crinoids – isocrinids (Isocrinida) have undergone a striking decline in shallow-sea environments and today they are restricted to deep-sea settings (below 100 m depth). However, the timing and synchronicity of this shift are a matter of debate. A delayed onset of MMR and/or shifts to a retrograde, low-predation community structure during the Paleogene in the Southern Ocean were invoked. In particular, recent data from the Southern Hemisphere suggest that the environmental restriction of isocrinids to the deep-sea settings may have occurred at the end of the Eocene around Antarctica and Australia, and later in the early Miocene in New Zealand. Here, we report the anomalous occurrence of the isocrinids in shallow nearshore marine facies from the middle Miocene of Poland (Northern Hemisphere, Central Paratethys). Thus, globally, this is the youngest record of shallow-sea stalked crinoids. This finding suggests that some relict stalked crinoids may have been able to live in the shallow-water environments by the middle Miocene, and further confirms that the depth restriction of isocrinids to offshore environments was not synchronous on a global scale.

## Introduction

Today, stalked crinoids, including isocrinids (Isocrinida), are restricted to the depth below 100 m (e.g. Refs.^1,2^). However, in the geologic past, especially during the Triassic and Jurassic, they were abundant in shallow-sea (nearshore and inner shelf) environments (e.g. Refs.^3^). In a seminal paper on bathymetric distribution of fossil isocrinids from Euramerica, Bottjer and Jablonski^4^ showed that the shift to deeper environments started by the Cretaceous and was completed by the Eocene. A variety of causal mechanisms for this onshore-offshore pattern has been suggested including bias toward onshore deposits of Mesozoic age^5^. Nevertheless, the most likely driver was increased diversification of durophagous predators especially fishes and grazing echinoids. This has been supported by observations of Recent crinoids, which show increased frequency of regenerated arms in shallower than in deeper sea settings^6^. Evidence of numerous bite marks and arm/pinnule regenerations on shallow-sea Mesozoic crinoids ascribed to predation is also consistent with this hypothesis (e.g. Refs.^7–9^).

**Figure 1:**
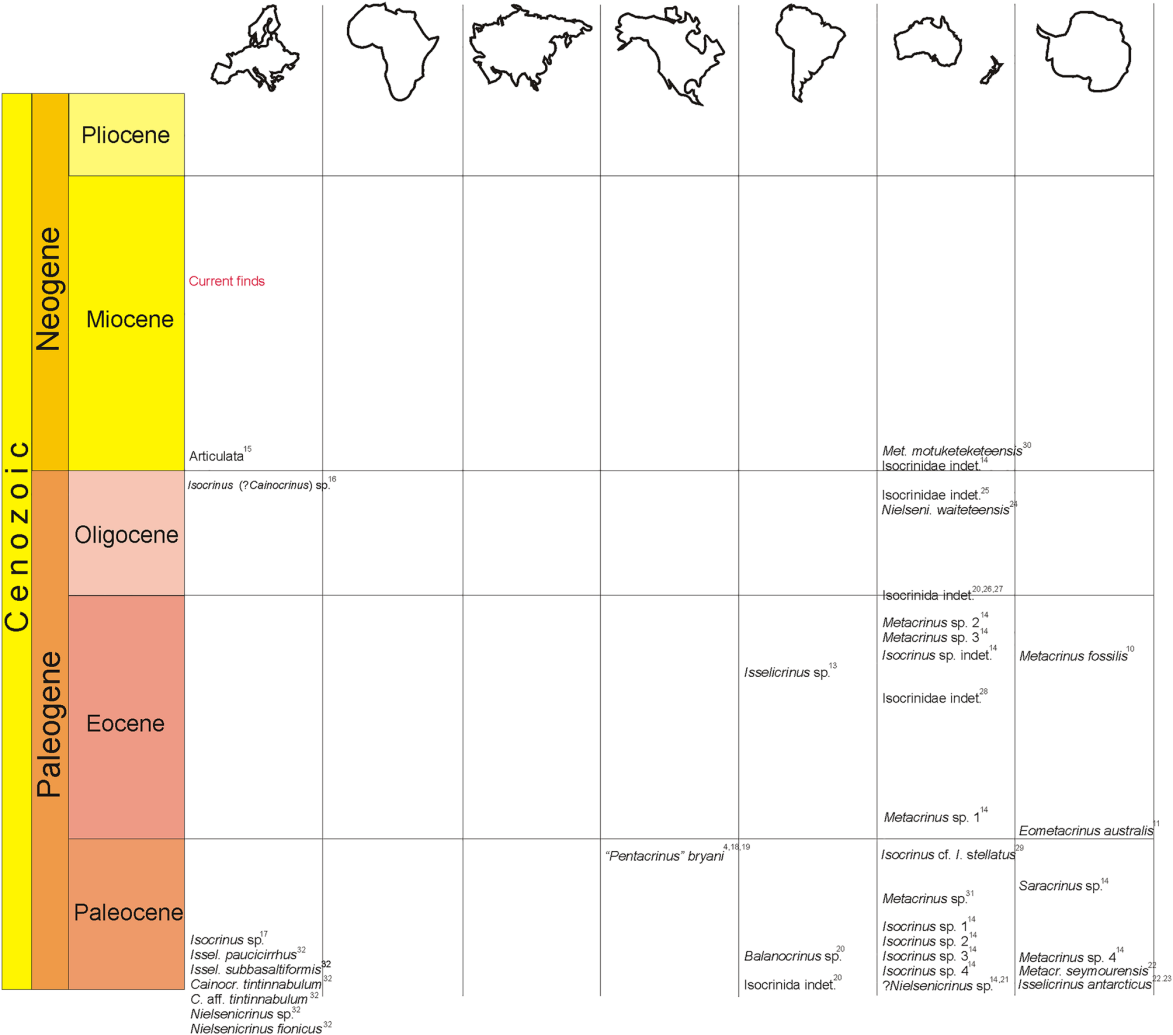
Cenozoic occurrences of shallow-sea stalked crinoids in different continents: Europe (Refs.^15–18^), North America (Refs.^4,19,20^), South America (Refs.^13,21^), Australia (Refs.^14,21–30^), and Antarctica (Refs.^10,11,14,31,32^). The depth of inner shelf is understood here as approximately 40 m (for details see Fig. 2.1. in Ribeiro^33^).

Admittedly, however, there are at least a few records of shallow-sea isocrinids from the Cenozoic, especially from the Southern Hemisphere (Fig. 1). Among the most spectacular occurrences are dense assemblages of ophiuroids and isocrinids in a late Eocene, shallow-marine setting of the La Meseta Formation on Seymour Island, Antarctic Peninsula^10–13^. This anomalous record has been referred by the latter authors to as the retrograde community structure that might have developed due to a combination of reduced predation pressure, a favorable temperature regime and increased upwelling in Antarctic surface waters. Nevertheless, a recent compilation on the Cenozoic occurrences of isocrinids from the Southern Hemisphere implies a continuous record of shallow marine isocrinids from the Cretaceous to the late Eocene, rather than temporal reversions^14^, suggesting that the timing of the onshore-offshore shift was not synchronous globally.

In this paper, we describe isocrinid ossicles from the middle Miocene shallow marine facies exposed in two localities in Poland. These findings not only represent the youngest global fossil record of shallow-sea isocrinids, but also support the view that occasionally, some stalked crinoids might have remained in the onshore environments long time after the initiation of the Mesozoic marine revolution.

### Geological settings and stratigraphy

**Figure 2:**
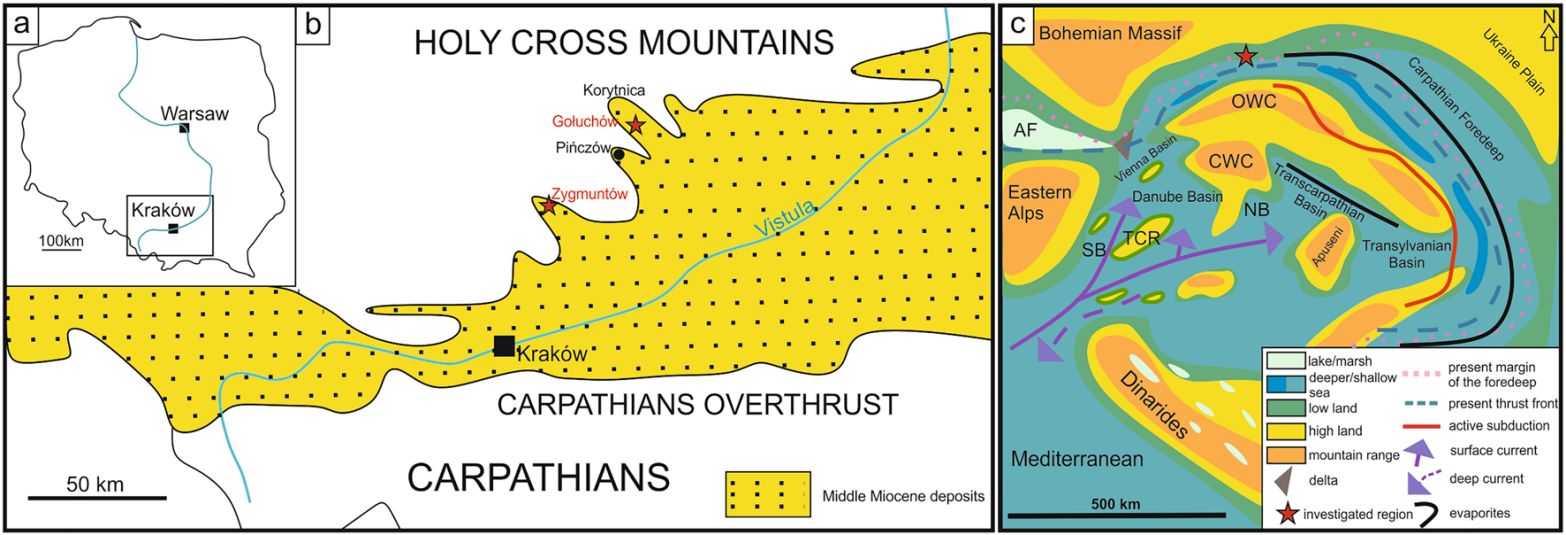
Location of the investigated outcrops. On the left, location sketch of the Gołuchów and Zygmuntów on the background of middle Miocene deposits in the southern Poland (**a**,**b**). On the right, palaeogeographic location of the studied outcrops ((AF) Alpine Foredeep; (CWC) Central Western Carpathians; (NB) Novohrad-Nógrád Basin; (OWC) Outer Western Carpathians; (SB) Styrian Basin; (TCR) Transdanubian High; (VB)) (**c**). Redrawn and modified after Studencka et al.^34^; Kováč et al.^35^.

Field works aimed at collecting crinoid material were conducted in two localities in the southern Poland (Fig. 2), namely Gołuchów and Zygmuntów (near Książ Wielki). Gołuchów is located on the southern edge of the Holy Cross Mountains, approximately 30 km south of Kielce city and approximately 20 km east of Jędrzejów city. The outcrop is located in a disused quarry (coordinates: 50.622703 N, 20.615972 E). Zygmuntów profile (near Książ Wielki) is located approximately 40 km southwest of Gołuchów and approximately 30 km south of Jędrzejów city. Herein, as a result of the construction of the S7 Cracow-Kielce expressway, Miocene sediments are exposed (coordinates: 50.26339 N, 20.11198 E).

Both localities are located in the marginal, northern part of the Carpathian Foredeep, on the southern foreland of the Holy Cross Mountains^36,37^ (Fig. 2). In the Miocene, this region was located in the northern part of Central Parathetys, which was characterized by the presence of numerous shallow marine bays^34,36,38–43^ (Fig. 2).

**Figure 3:**
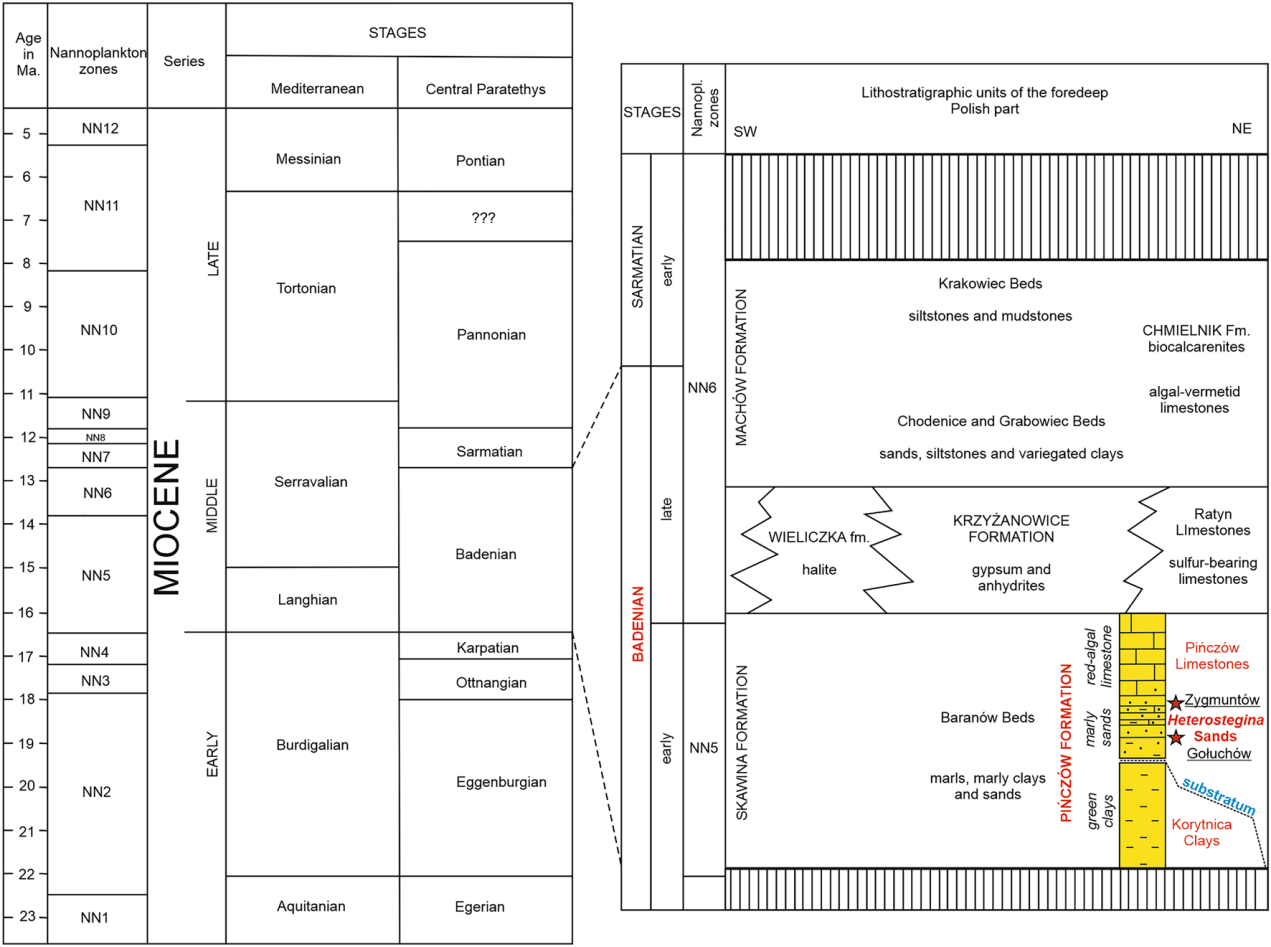
Stratigraphic scheme of the Miocene (after Steininger et al.^48^) and stratigraphic schemes for the foredeep depozone of the Fore-Carpathian Basin in Poland (modified after Jasionowski^49^; Oszczypko et al.^37^).

Stratigraphically, sediments exposed here belong to the lower part of the middle Miocene Mediterranean stages (Langhian and Serravalian) corresponding to the Central Paratethys Badenian stage (e.g. Ref.^35^; Fig. 3). In the local lithostratigraphic division, the studied sites are classified to the so-called Pińczów formation^42–45^, that is represented by siliciclastic, siliciclastic-carbonate and carbonate lithoral and sublithoral facies^36,38,39,42,45–47^(Fig. 3). Similar facies of this age continue in the eastwards towards Ukraine^34,47^.

In the studied area, depending on the palaeomorphology of the basement, various clays, sands, marly sands, sandy limestones and limestones overlie abrasive Upper Jurassic (Kimmeridgian) or Upper Cretaceous (Campanian–Maastrichtian) substrate^38,50^ (Fig. 3). According to the local lithostratigraphic unites, the sediments exposed in both localities belong to the so-called *Heterostegina* Sands (Fig. 3). Both with the so-called Korytnica Clays, these levels are included in the Badenian dinocyst Zone *Unipontidinium aquaeductum* equivalent of Nannoplankton Zone NN5-6^45,51–54^.

Within the sedimentary succession of the *Heterostegina* Sands, marly or calcareous sands and sandy limestone units yielding abundant fossils may be distinguished^38,39,41,45,46^. The most characteristic features for *Heterostegina* Sands are large benthic foraminifers (*Heterostegina* and *Amphistegina*)^53^, along with oval rhodoids and isolated colonies of red-algae (*Lithothamnium* in older literature) ^42,43,46^. Small and large-sized pelecypods, lingulid brachiopods, echinoids, starfish and large gastropods are abundant in these sediments^38,41,46,55^. Large oysters, commonly found herein, are typically encrusted by serpulids.

#### Gołuchów quarry

**Figure 4:**
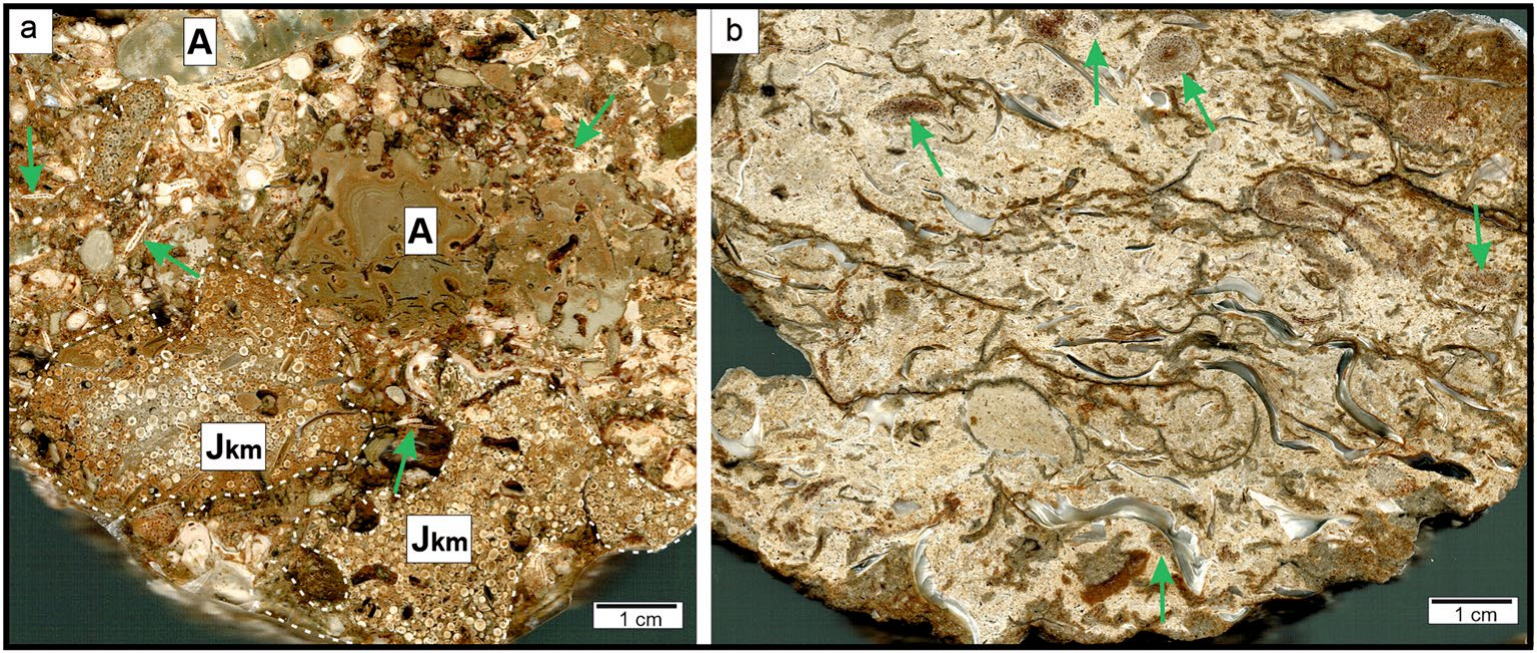
Polished slabs from Gołuchów Quarry. (**a**) Bioclastic floatstone/rudstone with numerous large Badenian benthic foraminifers (arrows), bivalve shells, bored algae (A) and oolitic clasts (Jkm). (**b**) bioclastic-bivalve floatstone with thick-shelled parallel or chaotic distributed bivalves and bryozoans (arrows). In calcareous matrix visible numerous small quartz grains.

At the base of the quarry, strongly lithified lower Kimmeridgian oolitic-bioclastic limestone facies are exposed^38^. Directly above the Kimmeridgian rocks, Badenian sediments, mostly represented by fine-grained red-algal sandy limestones with pebbles of the Kimmeridgian oolitic limestone, are present^38^ (Fig. 4a) Above, fine detritical sands and poorly lithified limy or marly sandstones^38^ of *Heterostegina* Sands are developed. Herein, abundant fossils (Fig. 4b) are present, including foraminifers, molluscs, bryozoans, serpulids, echinoderms (asteroids, echinoids and stalked crinoids, the latter group described herein), and teeth of fish.

**Figure 5:**
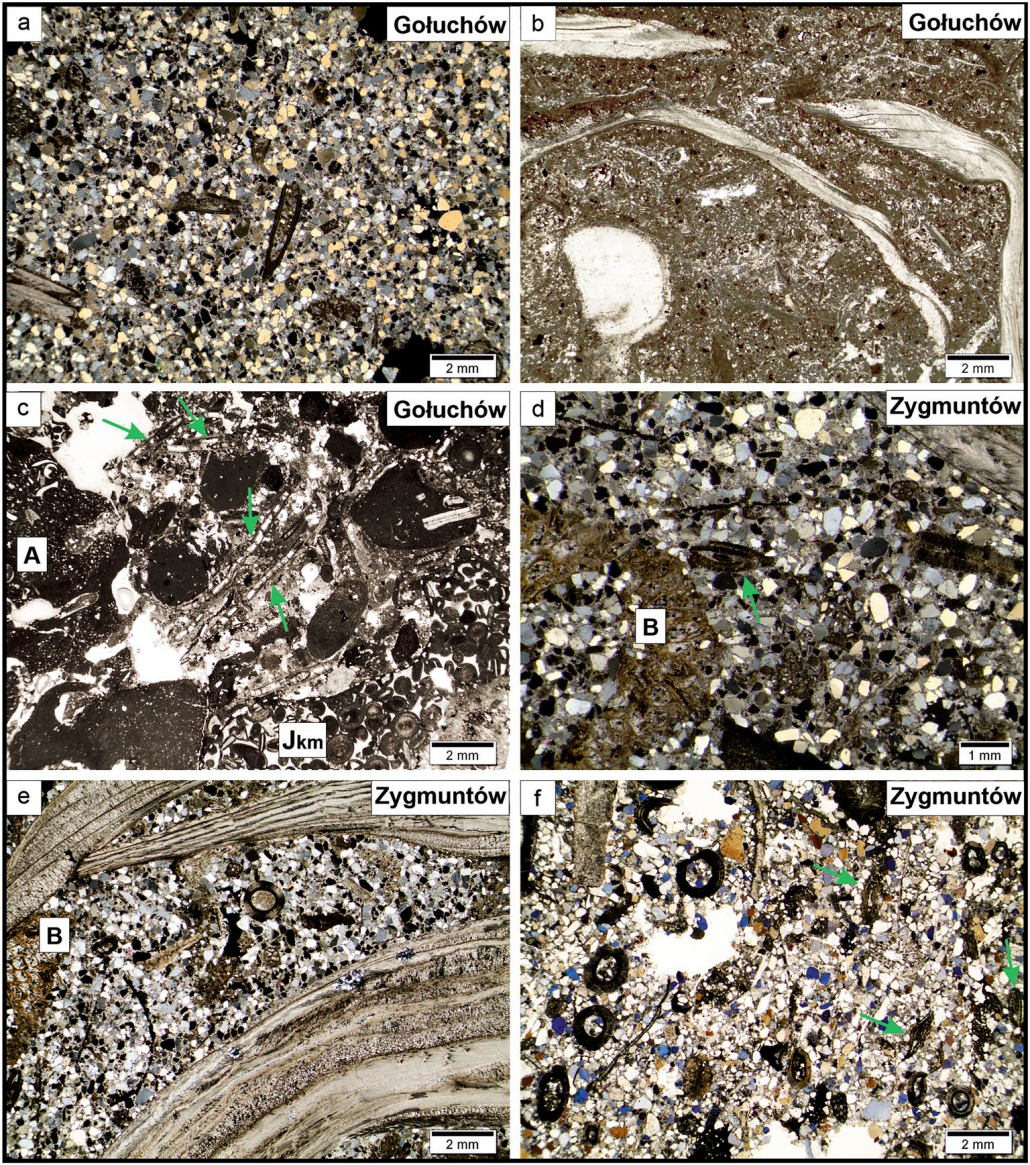
Microfacies from study oucrops. (**a**) bioclastic sandstone; visible mainly quartz grains, calcareous matrix and fragments of bivalves and polychaetes. (**b**) bivalve-bioclastic floatstone. (**c**) foraminifer-algae floatstone/rudstone with numerous large benthic foraminifers (arrows) and bored algaes (A); in the lower part clast of ooid limestone (Jkm). (**d**) bioclastic-reach sandstone with large benthic foraminifers (arrows), bryozoans (B), bivalves. (**e, f**) calcareous sandstone with numerous benthic foraminifers (arrows), bryozoans (B), shells of bivalves and polychaetes.

Our analyses of thin sections (Fig. 5a–c) taken from these rock levels suggest the presence of two facies types: (i) sandy limestone (FT 1-facies type 1) with numerous bioclasts (Fig. 5b,c) and (ii) sand and/or poorly lithified (sporadically marly) sandstone with numerous bioclasts (FT 2-facies type 2) (Fig. 5a). Numerous quartz grains and exclusively Miocene bioclasts embedded in the carbonate matrix are observed herein. FT 1 facies type is mostly represented by sandy limestone (rudstone-floatstone), within each numerous bored fragments of algal colony are present (Fig. 5b,c). Among bioclasts, abundant and well-preserved small and large benthic foraminifers (*Amphistegina* sp. and *Heterostegina costata*), molluscs, bryozoans and echinoderms were observed. The matrix is mostly composed of carbonates though small quartz grains are also observed. In the FT 2 sharp-edged quartz grains with a diameter of up to 1 mm, most often ~ 0.5 mm (Fig. 5a) are abundant. Glauconite grains are also common in these sediments. Among the bioclasts, Badenian small and large benthic foraminifers and polychaetes (Fig. 5a) were observed. Calcareous clasts up to 3 mm thick were also observed, in which fragments of red-algae and micritic sediments with small quartz grains resembling FT 1 can be distinguished.

#### Zygmuntów (near Książ Wielki)

In the newely exposed outcrop, sands with gravels embedded with two layers of clays, each with a thickness of about 0.6 m, and three layers of limestones, each 0.4 m thick, are present. Herein, within the lenses of sands and gravels, abundant benthic invertebrate fossils and fish teeth can be found. Crinoids were recorded in marly sands of the middle part of the section. Grill^56^ and Studencka^55^ based on characteristic bivalve assemblage suggested that these sediments belong to the Lagenidae Zone (early Badenian).

Our analyses of thin sections indicated that the bioclastic-reach sand and/or poorly lithified sandstone facies have limy matrix (Fig. 5d). Quartz grains typically have diameters of ~ 0.5 mm. Smaller grains are sharp-edged (Fig. 5e,f). Glauconite grains are also noted. Among numerous, exclusively Miocene bioclasts, we observed thick-shelled bivalves, bryozoans, serpulids, echinoids and fragments algal colonies (Fig. 5d–f). Abundant and well-preserved benthic foraminifers of various size (such as *Amphistegina* sp.) (Fig. 2f) are the most characteristic. Overall, this facies is similar to FT 1 observed in Gołuchów.

### Palaeoenvironmental interpretation

Facies and microfacies (Figs. 4, 5) recorded in both localities can be ascribed to the so-called *Heterostegina* Sands. Facies type 2 belongs to algal-amphisteginid/heterosteginid facies^44^ with numerous bryozoans and large benthic foraminifers. In general, these facies have been developed in near-shore shallow marine environment of moderate energy, that was occasionally subjected to storms, which resulted in faunal accumulations with coquina lags^41,46^. Mass occurance of well-preserved large foraminifers (*Amphistegina* and *Heterostegina*) are typical for shallow marine early Badenian Paratethys deposits. It is noteworthy that Recent representatives of *Amphistegina* prefer shallow-waters at a depth below 20 m^57^. No linear current sedimentary structures were observed, which is indicative of higher turbulance during storms. Previous studies based on analyses of the shallow-water fauna determined the palaeodepth of these sedimentary environment to be several to several dozen meters^36,38,39,42,46^.

## Results

### Fish fossil assemblage

Apart from collected crinoid fossils, a number of fish teeth were recorded (they will be described in a separate paper elsewhere). In the Gołuchów quarry, quite numerous fish teeth were recorded, most of which belong to teleost fish (above 70% collected specimens). All of them belong to the family Sparidae (stratigraphic range Eocene–Recent^58^). Less numerous are shark teeth, mainly belonging to the family Odontaspididae, including *Carcharias acutissima* (Oligocene–Pliocene^59,60^) and *Araloselachus* cf. *vorax* (Miocene^61^).

Assemblage of fish teeth from Zygmuntów (near Książ Wielki) is less abundant but more taxonomically diverse. It is dominated by shark teeth (68% of all specimens) belonging to at least four families. Teeth of *Otodus megalodon* (Miocene–Pliocene^62^), *Cosmopolitodus hastalis* (Miocene–early Pleistocene^59,60^), *Isurus* (Paleocene–Recent^59^) and *Galeocerdo* (Eocene–Recent^59^) were found here. Myliobatoid teeth representing the genus *Aetobatus* (Paleocene–Recent^5^) are occasionally found. Teleost fish teeth and tooth plates constitute 24% of collected teeth specimens, and are represented only by Sparidae.

No fish teeth from Mesozoic taxa were found at both sites. The stratigraphic ranges of all elasmobranch genera and teleost Sparidae are Cenozoic. Sparid fishes, whose teeth are present in Gołuchów are typically reef-associated forms and prefer warm and shallow water environments^63^. Sparid teeth are adapted to a durophagous diet and indicate feeding on shelled invertebrate fauna^58,60,63^.

### Crinoid description

**Figure 6:**
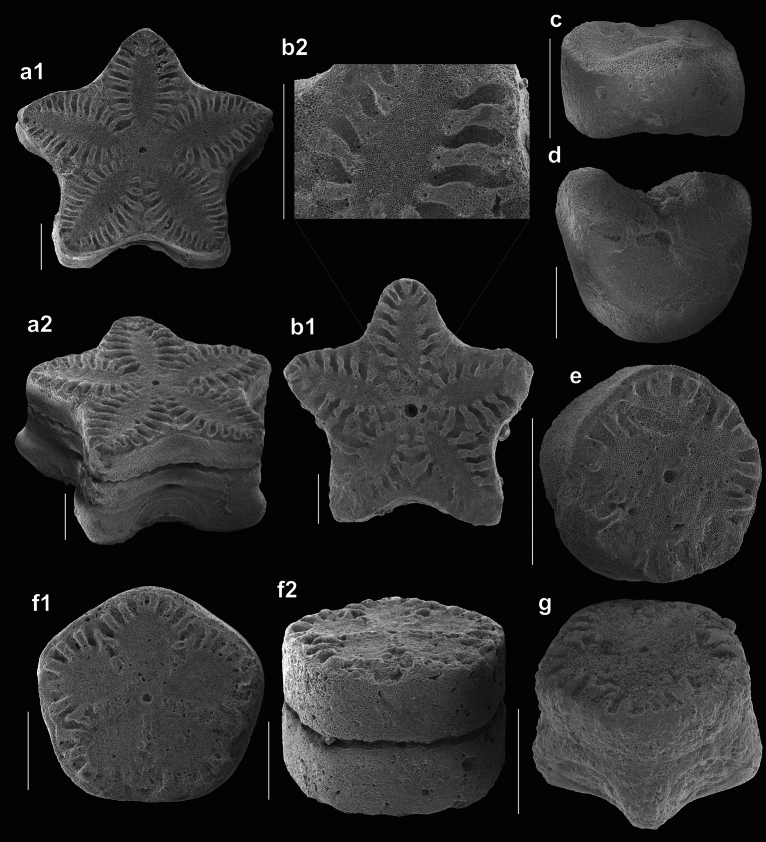
Metacrininae gen. et sp. indet. from Miocene (Badenian) strata of southern Poland. Scale bar equals 1 mm. (**a**) articular facet of proximal columnal (**a1**) and oblique view of proximal pluricolumnal (**a2**). Zygmuntów locality. GIUS 12–1475/8. (**b**) Articular facet of proximal columnal (**b1**) and enlargement of petal floor (**b2**). Zygmuntów locality. GIUS 12-1475/9. (**c**) oblique view of cirral. Gołuchów locality. GIUS 12-1475/3. (**d**) Proximal facet of secundibrachial. Gołuchów locality. GIUS 12-1475/4. (**e**) oblique view of distal columnal. Gołuchów locality. GIUS 12-1475/1. (**f**) articular facet of distal/medial columnal (**f1**) and oblique view of distal/medial pluricolumnal (**f2**). Gołuchów locality. GIUS 12-1475/5. (**g**) oblique view of distal/medial pluricolumnal. Gołuchów locality. GIUS 12-1475/7.

In both localities fragments of stems and isolated brachial of isocrinids were recorded (Fig. 6). Their detailed description is given below. Systematic description and terminology follows Hess and Messing^64^.

### Systematic palaeontology

Order Isocrinida Sieverts-Doreck, in Moore et al.^65^.

Suborder Isocrinina Sieverts-Doreck, in Ubaghs^66^.

Family Isselicrinidae Klikushin^67^.

Subfamily Metacrininae Klikushin^67^.

Metacrininae gen. et sp. indet.

#### Material

25 columnals and pluricolumnals consisting of up to 3 columnals, 1 secundibrachial and 1 cirral from Gołuchów locality, 5 columnals and 2 pluricolumnals consisting of up to 3 columnals from Zygmuntów (near Książ Wielki) locality (Fig. 6). The Miocene (Badenian) crinoids from southern Poland are housed at the University of Silesia in Katowice, Faculty of Natural Sciences, Institute of Earth Sciences (Poland), under the catalogue number GIUS 12-1475.

#### Measurements

Columnal diameter varies from 1.2 up to 4.7 mm; columnal height varies from 0.39 up to 1.1 mm. Cirral diameter is 1.42 mm and its length 1.82 mm. The brachial is 0.45 mm-wide and 2.18 mm-high.

#### Description

Distal columnals are circular and high, medial columnals are pentagonal to pentalobate, and proximal columnals are stellate in outline. Nodal columnals are distinctly higher than internodals. All nodal columnals are pentagonal with swollen interradii. Nodal diameter ranges from 1.22 to 1.49 of internodal diameter, and nodal height ranges from 14 to 36% of internodal diameter. Internodal columnals display symplectial facets with distinct marginal crenulae that may be V-shaped and fused adradially. Petal floors are rhombic in case of distal and medial columnals. They are discoidal and tear-shaped in proximal columnals. They are separated from each other by adradial crenulae belts, consisting of two parallel sets of very minute tubercles. Number of both marginal and adradial crenulae varies from 24 in circular distal columnals up to 80 in star-shaped proximal columnals. Almost all columnals possess large radial pores. They are circular, and typically surround petal floors. Latera is smooth and straight in distal and medial columnals; it is smooth and distinctly convex in proximal columnals. Lumen is small and circular. Cirrus scars of nodal columnals are of similar diameter, with a width ranging from 33 to 46% of the nodal diameter. Cirrus scar height ranges from 72 to 87% of nodal height. They are oval and may be proximally short. Secundibrachial is muscular and rounded aborally. Pinnule socket is visible. Cirrus is oval and its latera is smooth.

#### Discussion

The material described herein is strikingly similar to those described from the Miocene of France and ascribed to metacrinid crinoids^68^. Among the subfamily Metacrininae, three genera (*Metacrinus*, *Eometacrinus*, *Saracrinus*) are known according to Hess and Messing^64^, but Améziane et al.^69^ distinguished two genera (*Metacrinus*, *Saracrinus*) within this subfamily. Although *Saracrinus* is closely related to *Metacrinus*, Hess and Messing ^64^ based on opinions of Meyer and Oji^10^, Ameziane^70^ and Roux et al.^71^ decided to separate it from *Metacrinus.*

Typical *Saracrinus* has 4 primibrachials, among which primibrachials 1 and 2 are united by cryptosyzygy. Similarly in the case of *Metacrinus*, cryptosyzygy occurs between primibrachials 1 and 2. In the case of species with 7 primibrachials, cryptosyzygy also occurs between primibrachials 4 and 5 or 5 and 6. In the case of secundibrachials cryptosyzygy occur between secundibrachials 2 and 3 or 3 and 4 and also in more distal parts of arms. Other articulations in brachials are muscular. In the case of *Eometacrinus*, 5 primibrachials are known and synarthry is known between primibrachials 1 and 2, and cryptosyzygy between primibrachials 4 and 5. All of the above indicate that without an access to more complete (articulated) arms of Metacrininae, it is not possible to classify isolated metacrinitid stem material at the genus level. This led to the inclusion of the current material in Metacrininae.

It should be stressed that the material at hand differs strongly from the previously recorded crinoid taxa in the Mesozoic of Poland^72–78^.

#### Distribution

Paleogene (Eocene)–Holocene.

## Discussion

Discovery of isocrinid crinoids from the shallow marine facies of the middle Miocene age in the southern Poland (Northern Hemisphere, Central Paratethys) has a number of palaeoecologic implications. Although, the presence of isocrinid columnals from this region (Książ Wielki) has been already noted in 1934 by Krach^79^, they were never described nor illustrated, and the scientific value of their occurrences was not appreciated. Subsequently, for a long time the outcrop has been overgrown and was not assessable to palaeontological investigations. However, recent roadworks, which enabled us to collect new samples containing isocrinid columnals, allowed us to thoroughly describe these important fossils.

Although our material is mostly disarticulated (Fig. 6), and represented predominantly by stem fragments, its morphologic (resemblance to other well known Miocene isocrinid taxa) and taphonomic features (some of the material represented by articulated non-abraded pluricolumnals showing ornamentation and delicate stereom microstructure), combined with the sedimentologic and palaeogeographic context (restricted nearshore bay situated far away from the deeper slopes; see Fig. 2) suggest that these crinoids were paraautochthonous, i.e. they were not subject to significant transport and/or redeposition before final burial. Indeed, in-situ observations and tumbling experiments on Recent crinoids demonstrated that taphonomic features are a useful proxy in assessing autochthonous and allochthonous ossicles^80,81^. Those, which are subject to significant transport typically reveal altered (thinned or rounded) shape and broken stereom trabeculae with numerous shallow abrasion-induced grooves (wear scars). At least some of the material, especially from the Zygmuntów locality, does not reveal any of these features. Although the ossicles from Gołuchów, are not so well preserved – their disarticulation gradient is higher and some of them are slightly bioeroded/abraded – such features are typical in such high energy environment^4,82^. Finally, no evidence of post-diagenetic breakage on cleavage planes, which are indicative of reworking and redeposition from older rocks, were noted.

Overall, these data suggest that some relic isocrinid fauna might have been able to live in shallow marine environments by the middle Miocene. These findings expand the number of isocrinid occurrences in the Cenozoic shallow marine facies. Importantly, our compilation and critical evaluation of published data (Fig. 1) on the Cenozoic shallow marine isocrinids, suggest that isocrinids from Poland, were actually the latest stalked crinoids which were able to live in the nearshore environment. This is quite surprising given the fact that in such environment, a variety of predators are common. Indeed, from both localities numerous teeth of fish predators (see above) and cidaroid echinoids were recorded [represented by common, shallow water, species *Eucidaris zeamays*^83^]. One may speculate that these crinoids might have not been a preferential prey for some of these predators at that time – today, at least some representatives of fish groups recorded in our localities (Sparidae) are known to prey upon crinoids^84^. One the other hand, fossil isocrinids, like their Recent descendants, are known to possess a number of anti-predatory adaptations (regeneration, autotomy and crawling abilities, among the others)^85–88^, which, to some extent, might help them survive in such an environment.

Notwithstanding, the results presented here suggest that the disappearance of isocrinids from shallow marine environments was asynchronous on a global scale. Not only in the Gondwana Realm they remained in shallow water long after the initiation of the Mesozoic marine revolution, but in Euramerica, some relic populations of isocrinid fauna might have been also able to inhabit such environments.

## Materials and methods

Field works have been carried out in 2021 in Zygmuntów, and in 2023 in Gołuchów. Fossils, in particular crinoids, but also fish teeth, were initially searched in the field. At this stage, few of them have been collected. Additionally, bulk samples weighing 30 to 40 kg were taken from each lithological levels. In Gołuchów these were: red-algal sandy limestones with pebbles and fine-detrital sands or weakly compact calcareous and marly sandstones representing *Heterostegina* Sands, whereas in Zygmuntów, sands with gravel, clays and limestones were taken. Further work was carried out in the Palaeontological Laboratory of the Institute of Earth Sciences of the University of Silesia in Katowice. The marly samples were washed only with a stream of hot water and sieved through sieves with mesh diameters of 1.00 mm and 0.315 mm. Limestone samples were boiled in a Glauberian salt solution; after cooling, they were frozen. This process was repeated twice. The macerated rock was washed with hot water, sieved through sieves and dried at 180 °C. The obtained residue was examined for the presence of fauna using an Olympus SZX7-TR30 binocular. Crinoids were documented only in finely grained marly sands.

## Data Availability

All data generated or analysed during this study are included in this published article.
